# Varying Degrees of Ventricular Unloading in the Heterotopic Rat Heart Transplant Model Demonstrated by Magnetic Resonance Imaging

**Published:** 2014-12

**Authors:** Carolyn A. Carr, Daniel Ball, Damian J. Tyler, Andrew Bushell, Amelia Sykes, Kieran Clarke, Rhys D. Evans

**Affiliations:** 1 Department of Physiology, Anatomy and Genetics, University of Oxford, Sherrington Building, South Parks Road, Oxford OX1 3PT, U.K.;; 2 Nuffield Department of Surgery, University of Oxford, John Radcliffe Hospital, Oxford OX3 9DU, U.K.

**Keywords:** heart failure, heart transplant, heterotopic, magnetic resonance imaging, unloading

## Abstract

**Objective:**

Left ventricular assist device placement is an increasingly common treatment for cardiac failure, resulting in cardiac unloading and potentially reversing the remodelling changes seen in heart failure. A popular animal model for human ventricular unloading is the rodent heterotopic non-working heart transplant; the volume loading status of this preparation is important to interpreting the resulting reverse remodelling yet has not been previously investigated. This study was designed to assess the variability of left ventricular volume loading in the rodent transplant model.

**Methods:**

Heterotopic abdominal heart transplant was performed on syngeneic rats; high resolution cine magnetic resonance imaging was subsequently performed on the heterotopic transplanted hearts in anesthetised rats, after variable post-transplant recovery times, in order to assess ventricular loading status.

**Results:**

Highly variable left ventricular volume loading status was demonstrated, with some hearts exhibiting considerable ventricular filling and ejection.

**Conclusions:**

These observations call into question the assumption that studies using this model are consistently examining fully unloaded ventricles, and indicate the desirability of *in vivo* imaging of such hearts to quantify the degree of ventricular loading.

## INTRODUCTION

Heart failure is a major cause of mortality in the Western world and this has led to considerable efforts in understanding its pathoetiology and rationalising its treatment. It is characterised by failure to maintain adequate cardiac output, with resulting volume overloading of the cardiac ventricle. Treatment aims to offload the ventricle and decrease ventricular overfilling; in extreme cases cardiac output can be augmented by implantation of a ventricular assist device (VAD) which mechanically pumps blood from the ventricle or pulmonary vein into the aorta. This intervention has the benefit of “unloading” the ventricle, maintaining coronary perfusion whilst decreasing wall tension. Originally conceived as a “bridge” to transplantation, whereby the patient awaited availability of a donor organ, it became apparent in some cases that functional recovery of the ventricle had occurred during unloading with the VAD, permitting removal of the device and preventing the need for transplantation (1, 2). This observation was unexpected since, although multiple etiologies lead to cardiac failure, it was thought that the myocardial changes occurring in heart failure are irreversible. These include changes in trophic factors, ion handling and contractile machinery (3), and energetic metabolism (4), a pattern of alterations termed ventricular remodelling. However, a major insight into the underlying mechanism has arisen from observations in unloaded hearts, in which “reverse remodelling” is seen to occur. Reverse remodelling may not be simply a reversal of failure-induced remodelling: in particular, myocardial energy substrate metabolism, changes in which have been implicated as a primary causative factor in cardiac dysfunction and heart failure (5), has been shown to change profoundly in ventricular unloading in human VAD patients (6). The adult working myocardium normally utilises fatty acids (FA; present as both non-esterified FA and triacylglycerols) for ~ 70% of its energy requirements, with the remainder mostly provided by glucose (7); in ventricular unloading, there is decreased FA oxidation and increased glucose utilisation, a pattern closely resembling that seen in the fetal heart. Furthermore, the unloaded heart demonstrates a reversion to a “fetal programme” of gene expression (8, 9), in which fetal protein isoforms are expressed in preference to adult isoforms, and this is observed in genes coding metabolic proteins, enzymes and transporters.

**Figure 1:**
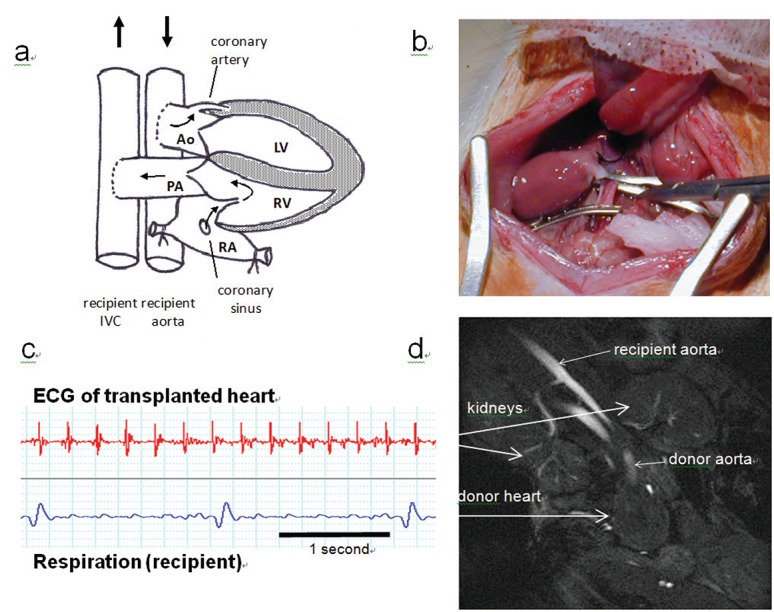
a, Rat heart heterotopically transplanted end-to-side onto recipient abdominal great vessels (Ao: aorta; PA: pulmonary artery; LV: left ventricle; RV: right ventricle: RA: right atrium). Myocardium is perfused and the heart beats but the left ventricular cavity should not receive blood and remain unfilled; b, Syngeneic heart transplanted into recipient abdomen. Cardiac ischemic time was < 75 min; c, Transplanted heart ECG detected by abdominal subcutaneous needles under anaesthesia for gating during subsequent MRI; d, Coronal MRI scan showing position of transplanted heart in relation to recipient kidneys, and blood flow in great vessels and transplanted aorta.

In an attempt to understand the changes (trophic factors, contractile machinery, ion handling and energetic metabolism) occurring in unloading and reverse remodelling, and to model the VAD-unloaded state of treated human heart failure, the rodent heterotopic heart transplant model has been advocated (10, 11), in which a donor rat heart is transplanted onto the abdominal great vessels of a recipient animal. The model has previously been extensively utilised in immunological studies of tissue rejection, where the donor heart can be transplanted either in “non-working” (unloaded) mode, with retrograde perfusion, or in “working” (loaded) mode with blood flow from the left atrium into the left ventricle (LV) (12-14). In ventricular unloading studies, however, syngeneic animals are used with the transplanted heart connected so as to receive coronary perfusion but no ventricular filling (unloaded; Fig. 1a) (15). The applicability and validity of this model depends on the ventricle remaining unloaded; however, to date this has not been examined. We hypothesised that volume loading status of the rat left ventricle following heterotopic transplant is variable, and can act as a potentially confounding factor in comparative loading-unloading studies based on this model. High field magnetic resonance imaging (MRI) has previously been used to study the function of hearts heterotopically transplanted in a variety of ventricular-loaded heart models (12-14, 16) with a view to detecting rejection of allogeneic donor hearts. In this report we describe a surprisingly high degree of variability in loading status and consequent wall motion in heterotopically transplanted “unloaded” hearts, detected using high resolution cine MRI.

## MATERIALS AND METHODS

### Animals

Experiments were performed in accordance with the United Kingdom Home Office guidelines under The Animals (Scientific Procedures) Act, 1986. Inbred male Fischer F344 rats (200-250 g) were obtained from a commercial breeder (Harlan, Bicester, UK) and maintained on a standard laboratory chow diet with a 12h light: 12h dark cycle and free access to water.

### Heterotopic heart transplant

Hearts were transplanted by one operator (AB) by the method of Ono & Lindsey (15); the procedure was performed using an operating microscope. Briefly, the recipient rat was anesthetised with inhaled isoflurane 2.0% v:v (Abbott Laboratories, Maidenhead, Berkshire, UK), and the abdominal vessels exposed; perforating lumbar vessels were temporarily ligated. The donor rat was then anesthetised, heparin (1000IU; Wockhardt UK Ltd, Wrexham, UK) was administered through the inferior vena cava (IVC), and the heart arrested with ice-cold potassium-cardioplegia solution (Plegivex; Ivex Pharmaceuticals, Larne, UK). The donor heart was excised and placed in ice-cold cardioplegia solution whilst the recipient rat great abdominal vessels were clamped. End-to-side anastomosis of the donor heart aorta to the recipient infrarenal abdominal aorta, and of the donor heart pulmonary artery to the recipient inferior vena cava was then performed using 10/0 nylon suture (BEAR surgical sutures, Kyowa Precision Instruments Co. Ltd., Chiba, Japan) (Fig. 1b). Clamps and lumbar vessel ties were removed, and the heart reperfused; spontaneous beating was observed in each case. Total cardiac ischemic time was <75 min, of which cold ischemic time was <45 min. The abdomen was closed and the animal recovered in a warmed box until conscious and moving freely. Animals were maintained for up to 24 days post-operatively with regular palpation of the transplanted heart pulse.

### Cardiac cine MRI

Cardiac cine MRI was performed at 6-24 days postoperatively using a 55 mm birdcage coil (Rapid Biomedical) in a vertical bore 500 MHz, 11.7 T MR system with a Bruker console running Paravision 2.1.1 (see Online Resource Supplementary Movies). Rats were anaesthetised with 2.0% v:v isoflurane in oxygen and positioned supine in a purpose built cradle. Two ECG signals were obtained, one from the recipient (thoracic) heart and a second, for gating, was obtained from the heterotopic transplanted heart using two subcutaneous needles adjacent to the palpated beat (Fig. 1c). The transplanted heart was located using scout images (Fig. 1d). To measure cardiac function, a stack of contiguous 1.5 mm true short axis ECG-gated cine images were acquired to cover the entire left ventricle. The imaging parameters were: field of view 51.2 × 51.2 mm; matrix size 256 × 256; TE/TR 1.43/4.6 ms; 17.5° Gaussian excitation pulse. The epicardial and endocardial borders were outlined in end-diastolic and end-systolic frames using the freehand drawing tool in Image J (17). Cardiac mass and left ventricular volumes were summed over the whole heart. For comparison, scans were also performed of untransplanted (orthotopic) control hearts under identical conditions of anaesthesia and positioning.

### Statistical analysis

Results are means ± SE [range]. Statistical analysis was performed by Student’s t test, and Spearman’s coefficient of rank correlation, as appropriate, using SPSS. Statistical significance was set at *P*<0.05.

## RESULTS

**Figure 2:**
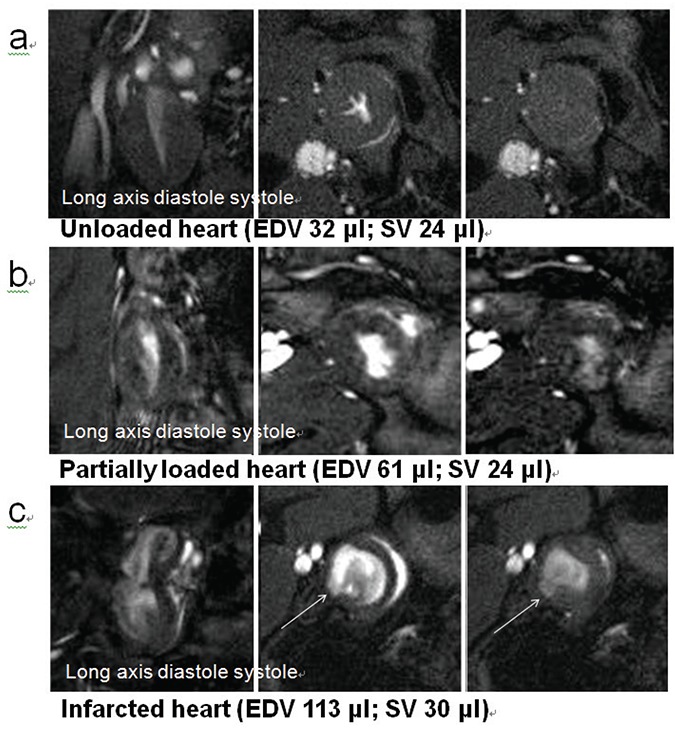
a, MRI cine frames (long axis; mid-ventricular short axis diastole; mid-ventricular short axis systole, slice frame 1.5mm; EDV: end-diastolic volume; SV: stroke volume) showing unloaded left ventricle; coronary venous effluent visible in right ventricle; b, as a, demonstrating partially loaded left ventricle; c, as a, demonstrating regional left ventricular wall motion abnormality (arrow) and paradoxical movement. SV calculated as EDV-ESV.

A total of 22 hearts were transplanted. All surgical procedures were performed by a single surgeon (AB) who has extensive experience of rodent (principally mouse) heterotopic heart transplants, (~20 years; ~3000 procedures) (18). During these experiments one recipient animal died following transplantation (bleeding; 5% mortality) and one transplanted heart failed to beat and was discarded (5%); the remaining beating heterotopic hearts (n=20) were studied (Table 1). The transplanted heart could be identified by MRI in the abdomen, below the kidneys in coronal section (Fig. 1d; Fig. 2). Good long and short axis views were obtained from all hearts, and blood could clearly be seen flowing through the right atrium before entering the right ventricle (Online Resource Supplementary Movie 1). Although all hearts could be readily detected by palpation, and were felt to be beating strongly, it was clear from cine MR images that the degree of left ventricular unloading varied substantially between individual hearts (Fig. 2a; Fig. 2b; Online Resource Supplementary Movies). Furthermore, in hearts which remained partially volume loaded with visible left ventricular filling and emptying, blood flow across the aortic valve could be clearly detected in long axis views (Online Resource Supplementary Movies 2a-4a). For comparison, in two additional animals not included in the quantitative analysis, transplantation of the heart was prolonged (ischemic time >100 min), causing ischemic damage to the myocardium and resulting in regional wall motion abnormality and ventricular dilatation; areas of infarcted myocardium were clearly visible (Fig. 2c; Online Resource Supplementary Movies 4a and 4b). Epicardial and endocardial borders were measured in end-diastolic and end-systolic frames by freehand drawing tool, and summed over the whole heart to estimate LV mass (184-558 mg), end-diastolic volumes (EDV; 19-139 ml) and end-systolic volumes (ESV; 2-93 ml); calculated stroke volumes (SV=EDV-ESV) varied from 10-56 ml and cardiac outputs (SV × heart rate) from 3-17 ml/min. Hearts were empirically divided into “unloaded” and “partially loaded” groups according to the LV end-diastolic volume (LVEDV): 12 of the 20 transplanted hearts studied (60%) had LVEDV less than 60 ml, whilst 8/20 of the transplanted hearts (40%) had LVEDVs of more than 60 ml (Table 1). The highest LVEDV recorded, 139 ml, was more than one third the mean control (loaded) LVEDV (380 ml). 40% of transplanted hearts (8/20) had a SV more than 10% (27 ml) of the average control (loaded) SV (270 ml). Following explantation, transplanted hearts were weighed and found to be significantly smaller than the native heart (transplanted heart mass 570 ± 39mg (n=20); native heart mass 1300 ± 92 mg (n=5) *P*<0.001), suggesting cardiac “hypotrophy”, but there was no correlation between stroke volume and cardiac mass (R2=0.235; *P*=0.32). Ventricular pressures were not measured in this study, and therefore no conclusions regarding workload can be made. There was also no correlation between the length of time post-surgery at which cardiac function was measured (6-24 days) and the observed degree of ventricular unloading (data not shown), so that differences in cardiac function were more probably due to variable surgical anatomy and its consequences.

**Table 1:** Hemodynamics of heterotopic heart transplant

Heart		n	LV EDV (ml)	LV SV (ml)	LV mass (mg)

Unloaded (LV EDV < 60 μl)	heterotopic transplanted	12	32 ± 3 [19-51]	19 ± 2 [10-28]	289 ± 26 [184-444]
Partially loaded (LV EDV > 60 μl)	heterotopic transplanted	8	84 ± 11 [61-139]***	33 ± 5 [14-56]**	315 ± 46 [171-558]
Loaded	orthotopic control	5	380 ± 19 [342-443]	270 ± 13 [246-311]	555 ± 35 [508-668]

22 rats underwent heterotopic (abdominal) heart transplant; 20 successful transplants were subsequently examined for left ventricular (LV) loading status by cine MRI. EDV: end-diastolic volume; SV: stroke volume. Loading status was empirically divided at 60 ml LV EDV. Control hearts were unoperated orthotopic hearts. For further details see text. Results are means ± SEM [range]. Statistically significant differences between the two heterotopic transplant groups are indicated:

** *P*<0.01;

*** *P*<0.001.

## DISCUSSION

Rodent heart transplantation models are increasingly used both in immunological studies of tissue rejection, and in studies of load-dependency of myocardial remodelling (usually as a model for the ventricular unloading seen in association with the use of ventricular assist devices). Transplanted hearts may be allogeneic or syngeneic and may be implanted as orthotopic grafts in the thorax or heterotopic grafts in the abdomen, pelvis or neck; in practice, the technical challenges of orthotopic cardiac transplantation in rodents have precluded this approach and therefore in practice heterotopic sites have been used. For immunological studies, a loaded heart is desirable, and many schemes have been described to ensure the “working” heterotopic heart is filling and ejecting (19); MRI has now confirmed that these hearts are indeed volume-loaded (12-14, 16). However, recent interest has focussed on the cardiac response (including myocardial metabolism) to ventricular unloading, principally in response to observations that the use of VADs in humans can lead to reversal of heart failure-associated remodelling, and hence improvement in the clinical condition, and obviate the need for subsequent intervention. In particular, the demonstration that profound metabolic changes occur in ventricular unloading, characterised by a reversion to a fetal programme of gene expression (8) in which substrate utilisation shifts from FA oxidation to glucose metabolism, has led to speculation that substrate selection could be intimately involved in the pathogenesis of heart failure and central to its recovery, and led to intense study of this phenomenon, using the transplanted heart as a model for the human situation. Hence, LV-unloaded heterotopic transplanted hearts are increasingly used in studies of the pleiotropic reverse remodelling effects of ventricular unloading as a model for human VADs in the management of heart failure, but this work is predicated on the assumption that the heterotopic transplanted heart is reliably unloaded. We have used high-resolution cine MRI to demonstrate that, contrary to expectations, the classic Ono and Lindsey technique (15) (in which there is no direct connection between inflowing blood and the left atrium, and hence left ventricle) may be associated with partial ventricular volume loading and measurable ventricular ejection. This is supported by the observation both of blood flowing through the left atrium and into the left ventricle, and of blood flowing out through the aortic valve (which is clearly observed to open) during ventricular systole. The hemodynamic origin of this flow is however not clear – high Thebesian blood flow may account for a significant degree of left ventricular filling, particularly in the rat (20), although it is unlikely that this could account for the left atrial filling. Aortic valve regurgitation in a partially loaded left ventricle is possible, and indeed surgically-induced aortic regurgitation prior to heterotopic transplantation of the rat heart has been advocated as a model of partial LV (un)loading, whereby the procedure increases LV volume loading, assessed by ultrasound, and induces LV chamber dilatation (21). From MRI movie images, it is likely that most of the LV loading and ejection seen in the current study was due to variable degrees of aortic valve regurgitation. It was not possible to establish the cause of the valve incompetency, but it may be due to variable aortic length, variable surgical anatomy, or myocardial ischemia; the latter seems unlikely in the majority of hearts reported here since regional wall motion was studied, and other than in the specific case described (Fig. 2c; Online Resource Supplementary Movie 4a and 4b), was not found. Consistent surgical technique may be predicted to minimise all three putative causes. It was not possible to accurately assess the competency of the mitral valve in these studies. Although LV pressure was not measured in the current study, and hence work could not be quantified, an estimate of cardiac work was attempted by measuring stroke volume; as with volume loading status, there was high degree of variability in ejection, and possibly therefore in workload. It is also likely that the observed partial loading accounts for the untypical pattern of atrophy/hypotrophy observed in such studies (22), although the putative relationship between contractile function and atrophic remodelling has been questioned (23, 24). It could be argued that partial loading of the left ventricle may be advantageous in a model of human VAD since the failing human heart typically remains partially loaded following ventricular assist placement, even at high VAD flow rates. Attempts have been made to address this with the development of “partially unloaded” (21, 25) and reloaded (26) heterotopic heart transplant models, but the “unloaded” technique remains the commonest model. Fully loaded transplant models are less variable in their loading characteristics (12-14), but preclude the possibility of studying the phenomenon of unloading and its impact on function; the possibility exists of using all these models experimentally in order to compare differing loading status, but this would absolutely require sophisticated assessment of the degree of loading prior to evaluation. The current findings suggest that LV volume loading status should ideally be assessed on an individual heart basis using an appropriate high resolution technique, such as MRI or echocardiography, that is capable of detecting aortic valve competency and the degree of ventricular filling *in situ*.
